# Adverse Clinical Outcomes among Inflammatory Bowel Disease Patients Treated for Urinary Tract Infection

**DOI:** 10.3390/jcm11051359

**Published:** 2022-03-01

**Authors:** Offir Ukashi, Yiftach Barash, Eyal Klang, Tal Zilberman, Bella Ungar, Uri Kopylov, Shomron Ben-Horin, Ido Veisman

**Affiliations:** 1 Department of Gastroenterology, Sheba Medical Center, Tel Hashomer, Ramat Gan 52620, Israel; bella.geyshis.ungar@gmail.com (B.U.); ukopylov@gmail.com (U.K.); shomron.benhorin@gmail.com (S.B.-H.); idoweiss37@gmail.com (I.V.); 2 Department of Internal Medicine A, Sheba Medical Center, Tel Hashomer, Ramat Gan 52620, Israel; 3 Sackler School of Medicine, Tel-Aviv University, Tel Aviv-Yafo 67011, Israel; yibarash@gmail.com (Y.B.); eyalkla@hotmail.com (E.K.); ztaltal@gmail.com (T.Z.); 4 Department of Diagnostic Imaging, Sheba Medical Center, Tel Hashomer, Ramat Gan 52620, Israel; 5 DeepVision Lab, Sheba Medical Center, Tel Hashomer, Ramat Gan 52620, Israel; 6 Infectious Disease Unit, Sheba Medical Center, Tel Hashomer, Ramat Gan 52620, Israel

**Keywords:** acute kidney injury, urinary tract infection, 30-day-recurrent hospitalization, Crohn’s disease, ulcerative colitis, inflammatory bowel disease

## Abstract

Background: Urinary tract infection (UTI) is the most common urologic complication among patients with inflammatory bowel disease (IBD). However, data regarding UTI outcomes in this population are scarce. We aimed to evaluate adverse outcomes of UTI among patients with IBD. Methods: This was a retrospective cohort study of consecutive adult patients who visited the emergency room (ER) at Sheba Medical Center due to a UTI between 2012 and 2018. Data included demographic and clinical variables. UTI cases were extracted using ICD-10 coding. Results: Of 21,808 (ER) visits with a UTI, 122 were IBD patients (Crohn’s disease—52, ulcerative colitis—70). Contrary to non-IBD subjects, patients with IBD had higher rates of hospitalization, acute kidney injury (AKI) and 30 day-recurrent hospitalization (59.3% vs. 68.9%, *p* = 0.032; 4.6% vs. 13.9%, *p* < 0.001; 7.3% vs. 15.6%, *p* = 0.001, respectively). Among patients with IBD, advanced age (*p* = 0.005) and recent hospitalization (*p* = 0.037) were associated with increased risk for hospitalization, while hydronephrosis (*p* = 0.005), recent hospitalization (*p* = 0.011) and AKI (*p* = 0.017) were associated with increased 30-day recurrent hospitalization. Neither immunosuppressants nor biologics were associated with UTI outcomes among patients with IBD. Conclusions: Patients with IBD treated for a UTI had higher rates of hospitalization, AKI and 30-day recurrent hospitalization than non-IBD patients. No association was observed between immunosuppressants or biologics and UTI outcomes.

## 1. Introduction

Inflammatory bowel disease (IBD) mainly encompasses two chronic inflammatory states primarily affecting the gastrointestinal (GI) tract: Crohn’s disease (CD) and ulcerative colitis (UC) [1,2]. The incidence of IBD in Western countries is stable and even falling, and the prevalence is estimated at 0.3% [3]. However, there is a rising rate of IBD incidence in newly industrialized countries. Thus, overall, the incidence and prevalence of IBD are increasing worldwide [2,3]. The national prevalence of IBD in Israel is 0.52% [4]. Considering the inflammatory nature of the disease, many agents used for IBD treatment alter immune system activity and potentially increase infection risk and infectious complications [5]. In addition, based on genome-wide association studies, the innate immune system might be impaired in IBD patients [5].

Urinary tract infection (UTI) is the most common bacterial infection, accounting for 1 million emergency room (ER) visits a year in the United States [6]. Among the main known risk factors to develop UTIs in the general population are anatomic abnormalities, diabetes, female sex and sexual activity [6]. Previous studies showed that UTI is the most common urologic complication among CD patients [7,8]. IBD features such as disease anatomic extent (e.g., entero-vesical fistulas [9]), disease duration, patient age and presence of urolithiasis [10,11] are highly associated with an increased risk of UTI among IBD patients. Furthermore, urolithiasis is considered one of the most frequent extraintestinal manifestations among IBD patients—with a prevalence of 8–19% (compared to only 0.1% in the general population) [12]. 

The available data regarding the outcomes of UTIs among patients with IBD are limited. We aimed to explore adverse outcomes of UTIs among patients with IBD. We also aimed to find predictors for adverse outcomes among this population.

## 2. Materials and Methods

### 2.1. Study Design and Patient Selection 

This was a retrospective, data-based cohort study that included adult patients who visited the ER at Sheba Medical Center due to a UTI between 2012 and 2018. Data were collected from an electronic repository of all ER visits and included tabular demographic and clinical variables and free-text physician records. The cohort consisted of patients with IBD and non-IBD patients. Any ER visit for anything except for UTIs was excluded. In addition, patients under 18 years old were excluded. For each patient, only the first ER visit for a UTI at Sheba Medical Center was considered, while the rest were excluded. IBD and UTI cases were extracted using the International Classification of Diseases (ICD-10) coding. 

### 2.2. Data Extraction 

The following data were collected from the electronic health record of Sheba Medical Center (Chameleon Electronic Medical Record):

Demographic factors—age (years) and sex.

IBD-related disease features—(disease extent by Montreal Classification and history of IBD surgeries).

IBD-related medications—amino salicylic acid (5-ASA) and similar agents, corticosteroids, immunomodulators such as azathioprine, mercaptopurine and methotrexate and biologics, including tumor necrosis factor alpha (TNF-α) inhibitors.

Background comorbidities (known to be associated with UTIs)—diabetes, benign prostate hyperplasia (BPH) (only for male sex), history of urologic tumors (kidney, bladder and prostate tumors) and history of urolithiasis. These medical conditions are well-known and distinctive risk factors for UTIs—leading to metabolic derangement and urine-flow impairment [5,12,13]. Other comorbidities were not extracted.

Microbiological features—blood and urine cultures (only for patients with IBD). Microbiological data regarding non-IBD patients were not available.

Other—history of hospitalization within three months prior to the index UTI ER visit and the presence of hydronephrosis (based on imaging modalities; ultrasonography or computed tomography scan).

### 2.3. Study Outcomes

1. Primary outcome—mortality within 30 days.

2. Secondary outcomes—hospitalization rate, hospitalization duration, acute kidney injury (AKI) and recurrent hospitalization for any reason within 30 days.

### 2.4. Data Analyses and Statistical Methods

Patients’ characteristics are presented as medians and interquartile ranges (IQRs) for continuous variables (not normally distributed), while categorical variables are expressed as proportions. Outcome comparisons between the cohort sub-groups (patients with IBD vs. patients without IBD and patients with CD vs. patients with UC) were performed using the Mann–Whitney test for continuous variables. The chi-square test or Fisher’s exact test were used for categorical variables. Demographic factors, background comorbidities, medications, IBD-related disease features and other variables were included in the univariable analyses related to each UTI outcome (as detailed above). Multivariable logistic regression was applied to identify independent predictors for each UTI outcome. Variables with *p* ≤ 0.1 on univariable analysis were included in the multivariable analyses. The backward selection method (Wald) was performed, and *p*-value > 0.1 was used as criteria for variable removal. All statistical tests were 2-sided, and *p*-value < 0.05 was considered as statistically significant. Statistical analyses were performed using SPSS software (IBM SPSS statistics for windows, IBM corp., Armonk, NY, USA).

## 3. Results

### 3.1. Patient Characteristics

**Figure 1:**
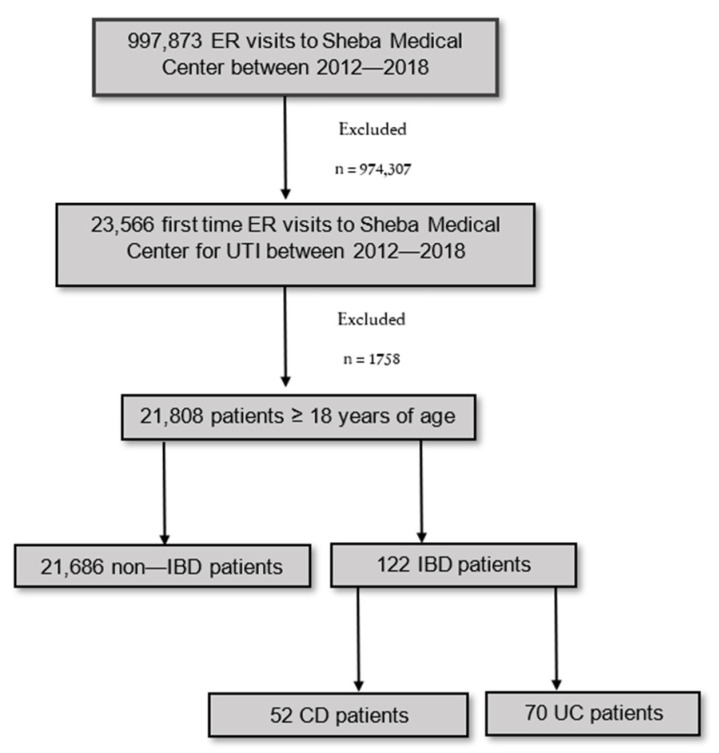
Study flow chart. Abbreviations: ER, emergency room; UTI, urinary tract infection; IBD, inflammatory bowel disease; CD, Crohn’s disease; UC, ulcerative colitis.

Out of 23,566 ER visits due to a UTI for the first time at Sheba Medical Center between 2012 and 2018, 1758 patients under 18 yo were excluded. The remaining 21,808 patients comprised the study cohort. Of them, 122 patients had a previous diagnosis of IBD (CD—52, UC—70), while there were 21,686 non-IBD patients (Figure 1). There were 6599 ER visits of IBD patients between 2012 and 2018; thus, the rate of UTI among IBD patients was 1.9% in this period of time. 

The baseline characteristics of patients with and without IBD are presented in Table 1. There was no difference in age (72.00 vs. 70.00 years, *p* = 0.351) or sex (*p* = 0.443) between the groups. BPH and urolithiasis were more prevalent among patients with IBD compared to patients without IBD (21% vs. 10%, *p* = 0.010; 11.5% vs. 3%, *p* < 0.001; respectively). On the other hand, urologic tumors and diabetes were slightly more common among the non-IBD group, but without statistical significance. The use of immunosuppressant and biological medications was more common among patients with IBD than patients without IBD. The IBD group had a higher rate of recent hospitalization (21% vs. 8%, *p* < 0.001), which was defined as hospitalization within three months prior to the index UTI ER visit. 

The characteristics of CD and UC patients are shown in Table 2. Urolithiasis and BPH rates were similar between the groups (12% vs. 11%, *p* = 0.985; 21% vs. 21%; *p* = 0.950, respectively). The use of 5-ASA was more common among the UC group (25% vs. 56%, *p* = 0.001), while TNF-α inhibitors (21% vs. 4%, *p* = 0.004) and azathioprine (15% vs. 3%, *p* = 0.013) were more commonly used among CD patients. In total, 18 (35%) patients of the CD group and 7 (10%) patients of the UC group had a history of previous abdominal surgery as part of the management of disease natural history (data were missing for 18 and 36 patients, respectively). The rate of missing data in regard to disease extent was 56% and 82% for CD patients and UC patients, respectively. 

### 3.2. Microbiologic Characteristics 

**Figure 2:**
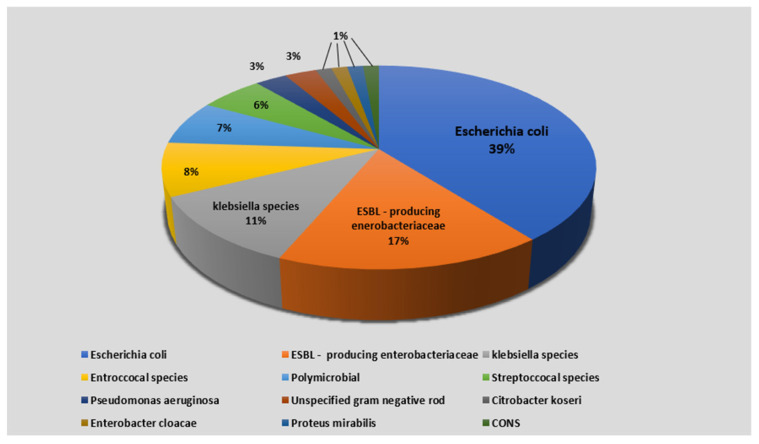
Frequency distribution of uropathogens grown in urine cultures among inflammatory bowel disease patients treated for UTIs. Abbreviations: ESBL, extended-spectrum beta-lactamase; UTI, urinary tract infection; CONS, coagulase-negative staphylococci. * Streptococcal species; other than enterococci.

Figure 2 depicts the frequency distribution of uropathogens among the IBD group. Of 122 IBD patients, only 110 patients had an available urine culture result. Of them, 71 patients had a positive urine culture, and 39 patients had a negative urine culture without any pathogen detection (the missing data rate for urine culture was less than 10%). The most detected bacterium was *E. coli* (39%). Extended-spectrum beta-lactamase (ESBL)-producing Enterobacteriaceae were grown on 17% of the urine cultures. Polymicrobial growth was detected in five urine cultures (7%). A total of 80 patients of the IBD group had an available blood culture result. Bacteria had grown on 13 of them, representing a positive blood culture (six—Escherichia coli; four—ESBL-producing Enterobacteriaceae, two—Klebsiella Pneumonia, one—Enterococcus Faecalis).

### 3.3. Outcomes and Predictors of Outcomes

The outcomes of UTI among the cohort population are summarized in Table 3. Patients with IBD had a higher hospitalization rate compared to patients without IBD (68.9% vs. 59.3%, *p* = 0.032), while no difference in hospitalization duration was observed between the groups. Though the mortality rate within 30 days was almost equal between the groups, patients with IBD had worse secondary outcomes, such as higher rates of AKI (13.9% vs. 4.6%, *p* < 0.001) and 30-day recurrent hospitalization (15.6% vs. 7.3%, *p* = 0.001). No statistical significance was demonstrated between CD and UC patients regarding the pre-defined UTI outcomes. Performing sub-analyses of UTI outcomes across different age groups, we discovered UTI outcomes to be comparable among patients ≥70 with or without IBD. On the other hand, patients with IBD under 70 yo had higher rates of hospitalization (59.3% vs. 44.5%, *p* = 0.030), AKI (16.7% vs. 1.7%, *p* < 0.001) and 30-day recurrent hospitalization (22.2% vs. 5.7%, *p* < 0.001) compared to patients without IBD at the same age. Notably, among patients under 70 yo, there was a trend toward a higher rate of 30-day mortality in favor of patients with IBD compared to patients without IBD (3.7% vs. 1.2%, *p* = 0.091). 

Table 4 and Table 5 show the results of univariable and multivariable analyses related to the pre-defined UTI outcomes. We found advanced age (adjusted odds ratio (AOR) 1.044, 95% confidence interval (CI) 1.013–1.076, *p* = 0.005) and a history of recent hospitalization (AOR 11.067, 95% CI 1.161–105.471, *p* = 0.037) to be independently associated with increased risk of hospitalization among patients with IBD treated for UTIs. Similarly, we learned that the presence of hydronephrosis (AOR 10.383, 95% CI 2.039–52.865, *p* = 0.005), recent hospitalization (AOR 4.494, 95% CI 1.420–14.221, *p* = 0.011) and AKI (AOR 4.683, 95% CI 1.325–16.548, *p* = 0.017) were independently associated with the increased probability of 30-day recurrent hospitalization. Using multivariable analyses, we did not find any of the examined variables to be associated with increased mortality rate within 30 days or with AKI. Notably, no association was observed between a history of previous abdominal surgery and UTI outcomes. 

## 4. Discussion

In this large, tertiary-center cohort, we examined the adverse outcomes of UTIs among patients with IBD compared to patients without IBD. We found higher rates of hospitalization, AKI and 30-day recurrent hospitalization in the IBD group compared to the non-IBD group. We also discovered advanced age and history of recent hospitalization to be associated with an increased risk of hospitalization among patients with IBD treated for UTIs. History of recent hospitalization and urological complications such as hydronephrosis and AKI were associated with an increased risk of 30-day recurrent hospitalization in this group. To the best of our knowledge, this study is the first to evaluate outcomes and predictors of outcomes among patients with IBD treated for UTIs.

Although both UC and CD mainly involve the GI tract, extraintestinal manifestations (EIMs) are common in both IBD phenotypes [14]. These manifestations can affect almost any organ system, including the urinary system. Urolithiasis, a well-known risk factor for UTIs [15], is common among IBD patients—8–19% compared to only 0.1% in the general population, with a higher risk in CD patients [12,16]. Moreover, disease anatomic features such as perianal involvement [11] and entero-vesical fistulas [9] are associated with a higher risk for UTIs among patients with CD. Surprisingly, we found that UTI outcomes were comparable between UC patients and CD patients. This finding may be explained by the small size of each subgroup or by the compatible rates of urolithiasis between the groups (Table 2), though a previous study by L. Peyrin-Biroulet [11] et al. showed no significant difference between the groups as well. However, further research will probably clarify this issue.

For the first time, our study demonstrated a higher rate of BPH in male patients with IBD compared to male patients without IBD [17]. Notably, no difference between baseline demographic characteristics (age and sex) of the IBD group compared to the non-IBD group was observed (Table 1). Next to urolithiasis, BPH is the most common cause of urinary outflow obstruction leading to UTIs [13]. Even though BPH was more prevalent among the IBD group, no association with adverse UTI outcomes among patients with IBD was demonstrated (Table 4 and Table 5). Thus, we could assume that BPH did not influence the worse outcomes among the IBD group. Interestingly, previous studies showed a high prevalence of prostate cancer among IBD patients [17,18]. However, in our cohort, we noticed a similar rate of urological tumors between the groups. Further studies should be performed to confirm an association between IBD and BPH, because this cohort might be biased by the selected diagnosis—UTI. Considering the high prevalence of BPH among the IBD group and the higher risk for hydronephrosis, obstructive AKI and UTIs among patients with BPH, it seems reasonable to screen IBD patients for this medical condition. Early diagnosis could allow appropriate management (e.g., alpha receptor antagonists [13]) to prevent unwanted adverse outcomes. 

Previous studies assessing the risk of AKI among patients treated for UTIs indicated that AKI occurs in 12.3–27.8% of the cases [19,20]. As mentioned, patients with IBD were more likely to develop AKI than patients without IBD. Not surprisingly, we found AKI as a predictor of increased risk for 30-day recurrent hospitalization rate. AKI is associated with short-term complications such as life-threatening electrolyte abnormalities (e.g., hyperkalemia), metabolic acidosis, fluid overload, and mechanical ventilation, the need for renal replacement therapy and even mortality (16.2–23.8%) [21,22,23]. Moreover, patients with AKI are more likely to develop chronic kidney disease (CKD), and patients who have CKD may rapidly progress to end-stage renal disease following an episode of AKI [24,25,26]. The dangers are overemphasized among patients with IBD who have a high prevalence of urolithiasis, as noted above [10]. Additionally, urolithiasis is one of the most common causes of hydronephrosis. Consequently, patients with IBD are at risk of hydronephrosis [27,28] and post-renal AKI [29]. BPH is another cardinal risk factor accounting for hydronephrosis [29]. Thus, it seems crucial to detect any renal impairment early and, in particular, urine outflow obstruction in these patients. In view of the virtues of ultrasonography (high availability, lack of ionized exposure risk and its usefulness in the detection of flow obstruction [30,31]), we think that it is reasonable to perform a urinary system ultrasonography for each IBD patient admitted due to a UTI. Additionally, supporting the fluid status is an important measure to maintain normal renal function among patients with IBD treated for UTIs [22,23,32]. 

This study demonstrated a higher rate of hospitalization among the IBD group treated for UTI. Our findings are consistent with previous studies—J. Burisch et al. [33] described a higher rate of all-cause hospitalization among IBD patients, particularly in the early years from diagnosis. Additionally, in a previous study by our group about IBD patients treated for pneumonia, we found a higher rate of hospitalization, though no difference in adverse outcomes was observed [34]. This study indicates that patients with IBD who had had a history of recent hospitalization for any reason prior to a UTI episode had an increased risk of hospitalization and 30-day recurrent hospitalization for any reason. It may be explained by the various complications associated with hospitalization state (e.g., nosocomial infection [35,36], AKI [37], deep vein thrombosis [38], etc.) that may lead to a further one. 

IBD treatments including TNF-α inhibitors, corticosteroids and immunomodulators alter the immune system, leading to an increased risk for an infectious disease [39]. It is possible that these characteristics are related to the results demonstrated in this study (Table 3). However, we did not find a significant association between IBD treatment agents and the primary or secondary outcomes (Table 4 and Table 5). The latter may be explained by a modest sample size; otherwise, it is conceivable that other risk factors had a more significant impact on the prognosis. Moreover, corticosteroid use patterns vary among IBD patients (e.g., continuous, intermittent or short-term, as needed by the patient [40]) and reliable follow up is often unavailable. In our study, we could not trace those patterns based on its retrospective design. Further prospective research which will address this issue is needed to better explore it. We could not examine the effect of Vedolizumab on UTI outcomes, because no patient in the IBD group had been treated with it, based on retrospective data extraction. Further research may include Tofacitinib and Ustekinumab medications which had not commonly been used in our IBD group between 2012 and 2018.

Our study has some limitations. First, UTI cases were extracted using the ICD-10 coding system based on the electronic records. Typing errors might have contaminated the study population with other diseases (e.g., UTI cases that have been categorized as non-UTI cases by typing mistakes, and vice versa). This is true also in cases where the physician has not documented a precise diagnosis due to a lack of attention to this issue. Second, due to the retrospective design of this study, data regarding blood cultures and urine cultures of patients without IBD were not available; thus, we could not make a reliable comparison of microbiological characteristics between the IBD group and the non-IBD group at the same period of time and under the influence of the same regional antibiotic resistance profile. Therefore, we could not estimate the association between IBD state and the probability to have bacteremia during urinary tract infection episode, among other contributing factors. Third, we could not evaluate the association between IBD-related features and UTI outcomes, probably because of the small sample size in each subgroup (e.g., CD group and UC group); this was accentuated by substantial missing data regarding disease extent due to the retrospective nature of this study. Disease extent by Montreal Classification was extracted from free-text summaries following gastroenterologist visits. Because most of the IBD patients in the cohort were treated out of the gastroenterology department at Sheba Medical Center, this information was not available. Fourth, the IBD group in this study was relatively small. A larger sample size may have allowed us to better investigate predictors of mortality and other outcomes of UTI among patients with IBD. Fifth, as a tertiary medical center, patients who are referred to our ER may have a worse illness compared to community-treated patients. This selection bias might have an influence on the study outcomes, but because the whole cohort (patients with and without IBD) was composed of patients who were referred to our ER, and the comparison was between the above-mentioned populations, we think it was well-balanced. Sixth, as mentioned above, we could not examine the effect of Vedolizumab, Tofacitinib and Ustekinumab on UTI outcomes, because no patient was treated with these agents during the study period. Seventh, the prevalence of ER visits for UTIs was around 2% of all ER visits, among both IBD and non-IBD groups; this is lower than the results of a previously published study, which demonstrated that UTI cases account for 3.3–4% of the hospitalizations [11] of IBD and non-IBD patients. We think that conceivable explanations for that is the different denominator (ER visits vs. admissions) and the different local epidemiologic features. Unfortunately, due to the retrospective design of this study, data regarding the hospitalization rate among all-causes hospitalization in our institute were not available. Eighth, because of the retrospective design of this study, data regarding potential confounding factors might have been missing. 

## 5. Conclusions

This is the first study to evaluate clinical outcomes among patients with IBD treated for UTIs. We demonstrated that in this population, UTIs encompass a greater risk for hospitalization, AKI and re-hospitalization within 30 days. Therefore, renal function monitoring, fluid maintenance and performing a renal ultrasonography scan to eliminate the existence of obstructive impairment should be considered. Notably, neither immunosuppressants nor biologics were found to influence UTI outcomes among IBD patients.

## Figures and Tables

**Table 1:** Baseline characteristics: IBD patients vs. non-IBD patients.

	IBD Patients (*n* = 122)	Non-IBD Patients (*n* = 21,686)	*p*-Value
Age(y)—median (IQR)	72.00 (49.75–83.00)	70.00 (51.00–82.00)	0.351
Male (%)	52 (43%)	9996 (46%)	0.443
**Comorbidity (%)**			
Diabetes (%)	20 (16%)	4353 (20%)	0.311
Benign prostate hyperplasia (%) ^$^	11 (21%)	1043 (10%)	0.010
Urolithiasis (%)	14 (11.5%)	623 (3%)	<0.001
Urologic tumor (%)	2 (2%)	588 (3%)	0.467
**IBD medications (%)**			
Amino salicylic acid and similar agents (%)	52 (42%)	12 (<1%)	<0.001
Corticosteroids (%)	21 (17%)	998 (5%)	<0.001
Azathioprine (%)	10 (8%)	1 (<1%)	<0.001
Methotrexate (%)	3 (3%)	113 (<1%)	0.003
Tumor necrosis factor alpha inhibitors (%)	14 (12%)	3 (<1%)	<0.001
**Miscellaneous (%)**			
Recent hospitalization (<3 months) (%)	26 (21%)	1790 (8%)	<0.001

^$^ Benign prostate hyperplasia proportions and *p*-values were only calculated for men. Abbreviations: y, years; IQR, interquartile range; IBD, inflammatory bowel disease.

**Table 2:** Baseline characteristics: Chron’s disease (CD) patients vs. ulcerative colitis (UC) patients.

	CD Patients (*n* = 52)	UC Patients (*n* = 70)	*p*-Value
Age(y)—median (IQR)	61.50 (37.50–79.50)	75.00 (65.75–84.25)	0.001
Male (%)	24 (40%)	28 (46%)	0.497
**Comorbidity (%)**			
Diabetes (%)	5 (10%)	15 (21%)	0.081
Benign prostate hyperplasia (%) ^$^	5 (21%)	6 (21%)	0.950
Urolithiasis (%)	6 (12%)	8 (11%)	0.985
Urologic tumor (%)	0 (0%)	2 (3%)	0.219
**IBD medications (%)**			
Amino salicylic acid and similar agents (%)	13 (25%)	39 (56%)	0.001
Corticosteroids (%)	11 (21%)	10 (14%)	0.320
Azathioprine (%)	8 (15%)	2 (3%)	0.013
Methotrexate (%)	2 (4%)	1(1%)	0.394
Tumor necrosis factor alpha inhibitors (%)	11 (21%)	3 (4%)	0.004
**Miscellaneous (%)**			
Recent hospitalization (<3 months) (%)	9 (17%)	10 (14%)	0.682

Abbreviations: y, years; IQR, interquartile range; IBD, inflammatory bowel disease. ^$^ Benign prostate hyperplasia proportions and *p*-values were only calculated for men.

**Table 3:** Urinary tract infection outcomes.

	IBD Patients	Non-IBD Patients	*p*-Value	CD Patients	UC Patients	*p*-Value
**30-day mortality (%)**	7 (5.7%)	1106 (5.1%)	0.750	3 (5.8%)	4 (5.7%)	0.990
**Hospitalization (%)**	84 (68.9%)	12,863 (59.3%)	0.032	37 (71.2%)	47 (67.1%)	0.636
**Hospitalization LOS median (d) median (IQR)**	3.00 (1.00–5.75)	3.00 (1.00–5.00)	0.990	3.0 (1.0–5.5)	3.0 (2.0–6.0)	0.251
**30-day r** **ecurrent hospitalization (%)**	19 (15.6%)	1591 (7.3%)	0.001	9 (17.3%)	10 (14.3%)	0.649
**Acute kidney injury (%)**	17 (13.9%)	998 (4.6%)	<0.001	10 (19.2%)	7 (10.0%)	0.145

Abbreviations: IBD, inflammatory bowel disease; CD, Chron’s disease; UC, ulcerative colitis; d, days; IQR, interquartile range; LOS, length of stay.

**Table 4:** Univariable and multivariable analyses: odds ratio and adjusted odds ratio for 30-day mortality and hospitalization among IBD patients treated for urinary tract infection.

Outcome	30-Day Mortality				Hospitalization			
Analysis	Univariable	Multivariable ^$^			Univariable	Multivariable ^$^		
	*p*-Value	AOR	95% CI	*p*-Value	*p*-Value	AOR	95% CI	*p*-Value
**Age**	0.126				0.034	1.044	1.013–1.076	0.005 ^^^
**Sex**	0.990				0.268			
**IBD disease**	0.990				0.636			
**Previous SHx**	0.615				0.534			
**5-ASA**	0.990				0.938			
**Corticosteroids**	0.412				0.779			
**Azathioprine**	0.545				0.427			
**Methotrexate**	0.665				0.934			
**TNF-alpha inhibitors**	0.327				0.315			
**Diabetes**	0.228				0.239			
**BPH**	0.616				0.283			
**Urolithiasis**	0.327				0.404			
**Urologic tumors**	0.725				0.338			
**Positive blood culture**	0.060	5.90	0.75–46.43	0.091	0.049			
**Hydronephrosis**	0.470				0.698			
Recent hospitalization *	0.152				0.050	11.067	1.161–105.471	0.037 ^^^
**Hospitalization**	0.067				-			
**Acute kidney injury**	0.978				0.063			
**Hospitalization LOS**	0.225				-			

* Recent hospitalization (<3 months). ^$^ Only variables that were included in the last step of the backward logistic regression analysis are presented in the table. ^^^ Variables which were found to be statistically significant in multivariable analysis. Abbreviations: AOR, adjusted odds ratio; CI, confidence interval; IBD, inflammatory bowel disease; SHx, surgical history; BPH, benign prostate hyperplasia; LOS, length of stay; TNF-alpha; tumor necrosis factor-alpha; 5-ASA; 5-Amino salicylic acid and similar agents.

**Table 5:** Univariable and multivariable analyses: odds ratio and adjusted odds ratio for acute kidney injury and 30-day recurrent hospitalization among IBD patients treated for urinary tract infection.

Outcome	Acute Kidney Injury				30-Day Recurrent Hospitalization			
Analysis	Univariable	Multivariable ^$^			Univariable	Multivariable ^$^		
	*p*-Value	AOR	95% CI	*p*-Value	*p*-Value	AOR	95% CI	*p*-Value
**Age**	0.634				0.096			
**Sex**	0.354				0.579			
**IBD disease**	0.145				0.649			
**Previous SHx**	0.238				0.108			
**5-ASA**	0.025	0.271	0.072–1.013	0.052	0.118			
**Corticosteroids**	0.151				0.629			
**Azathioprine**	0.184				0.687			
**Methotrexate**	0.326				0.390			
**TNF-alpha inhibitors**	0.389				0.154			
**Diabetes**	0.578				0.452			
**BPH**	0.627				0.534			
**Urolithiasis**	0.093				0.154			
**Urologic tumors**	0.566				0.540			
**Positive blood culture**	0.289				0.264			
**Hydronephrosis**	0.904				0.005	10.383	2.039–52.865	0.005 ^^^
Recent hospitalization *	0.810				0.016	4.494	1.420–14.221	0.011 ^^^
**Hospitalization**	0.063				-			
**Acute kidney injury**	-				0.016	4.683	1.325–16.548	0.017 ^^^
**Hospitalization LOS**	0.007	1.089	0.993–1.193	0.070	0.224			

* Recent hospitalization (<3 months). ^$^ Only variables that were included in the last step of the backward logistic regression analysis are presented in the table. ^^^ Variables which were found to be statistically significant in multivariable analysis. Abbreviations: AOR, adjusted odds ratio; CI, confidence interval; IBD, inflammatory bowel disease; SHx, surgical history; BPH, benign prostate hyperplasia; LOS, length of stay; TNF-alpha; tumor necrosis factor-alpha; 5-ASA; 5-Amino salicylic acid and similar agents.

## Data Availability

Not applicable.
